# Expressive writing intervention on optimism and coping modes of newly diagnosed people with human immunodeficiency virus: A randomized controlled trial

**DOI:** 10.1097/MD.0000000000048936

**Published:** 2026-05-29

**Authors:** Juan Tan, Shiping Feng, Qian Wen, Peisha Du, Chuntao Wu, Jing Yang

**Affiliations:** a Department of General Surgery, Public Health Clinical Center of Chengdu, Chengdu City, Sichuan, China.

**Keywords:** coping, expressive writing, HIV, optimism

## Abstract

**Background:**

To investigate the effect of expressive writing intervention on optimism and coping modes of newly diagnosed people with human immunodeficiency virus (HIV).

**Methods:**

A total of 84 newly diagnosed people with HIV in the care clinic of Public Health Clinical Center of Chengdu were randomly and equally divided into 2 groups with 42 patients in each group for a randomized controlled trial. The control group received 8-week regular health education and psychological guidance. The test group received an 8-week expressive writing intervention in addition to regular health education and psychological guidance. The life orientation test revised (LOT-R) and the medical coping mode questionnaire (MCMQ) were used to evaluate the optimistic tendency and coping modes of newly diagnosed people with HIV, respectively. The trial protocol and all amendments were approved by the Institutional Review Board of Public Health Clinical Center of Chengdu (PJ-K2020-36-01). This study was registered in the Chinese Clinical Trial Registry with registration (ChiCTR2200064585).

**Results:**

After the intervention, significant differences were found in the scores of LOT-R and MCMQ between the 2 groups (*P* <.05). Before and after the intervention, no significant difference was found in the scores of LOT-R and MCMQ in the control group (*P* >.05), but significant differences were found in the scores of LOT-R and MCMQ in the test group (*P* <.05).

**Conclusion:**

The expressive writing intervention would improve optimism and promote more positive coping modes for newly diagnosed people with HIV.

## 1. Introduction

Approximately 40.8 million individuals were living with human immunodeficiency virus (HIV) globally by the end of 2024, with an estimated 1.3 million new infections reported during the same year.^[1]^ In China, surveillance data from the Chinese Center for Disease Control and Prevention indicated 1.355 million people living with HIV/acquired immunodeficiency syndrome (AIDS) and roughly 100,000 newly diagnosed cases.^[2]^ Although HIV/AIDS remains incurable, the widespread implementation of antiretroviral therapy (ART) has markedly extended the life expectancy of affected individuals, rendering it comparable to that of the general population in many cases.^[3]^

Nevertheless, HIV infection exerts profound impacts beyond the physical domain, substantially influencing psychological well-being and social functioning. Newly diagnosed individuals face distinct psychosocial challenges as they grapple with the dual burden of disease-related distress and external pressures such as stigma, discrimination, and financial strain. These adversities often precipitate emotional disturbances, including self-denial, anger, feelings of inferiority, anxiety, and fear. Persistent negative emotions can hinder psychological adjustment, diminish quality of life, and compromise treatment adherence.^[4]^ Therefore, identifying and implementing evidence-based interventions that promote positive psychological adaptation and strengthen optimism and coping capacities is of critical importance for this population.

Positive psychology, conceptualized by Seligman and colleagues in the late 20th century, underscores the development of individual potential, positive affect, and adaptive psychological resources.^[5]^ Central to this framework is the notion that strengthening intrinsic capacities – such as optimism, hope, and perceived social support – enhances individuals’ resilience and adaptability when confronted with adversity. Optimism, a pivotal construct within positive psychology, has been empirically shown to buffer psychological stress, mitigate depressive symptoms, and facilitate the adoption of health-promoting behaviors.^[6]^ Among individuals with chronic illnesses, elevated levels of optimism are consistently associated with greater adherence to antiretroviral therapy, superior immunological outcomes, and higher life satisfaction.^[7]^ Despite these benefits, limited research has addressed effective approaches to cultivating optimism among people newly diagnosed with HIV.

As positive psychology has evolved, expressive writing has emerged as a thematic intervention strategy, particularly emphasizing the written articulation of positive emotions. This technique encourages individuals to write about, recall, and reflect upon positive experiences, thereby enabling them to process and express their inner thoughts and emotions.^[8]^ Writing about positive content has been found to elicit positive affect, broaden cognitive and attentional scope, and promote exploratory engagement with the environment.^[9]^ These processes, in turn, may facilitate cognitive restructuring, adaptive coping, and the development of enduring positive cognitive schemas.

Evidence from studies involving patients with breast cancer, colorectal cancer, and rheumatoid arthritis indicates that expressive writing can produce substantial psychological and physiological benefits.^[10]^ However, research examining its application in people living with HIV remains scarce. Accordingly, the present study employs a positive psychology–oriented expressive writing intervention for individuals newly diagnosed with HIV, with the objective of enhancing optimism, fostering adaptive coping mechanisms, and improving adherence to antiretroviral therapy.

## 2. Methods

### 2.1. Study design

This study was a randomized controlled trial. The trial protocol and all amendments were approved by the Institutional Review Board of Public Health Clinical Center of Chengdu (PJ-K2020-36-01). This study was registered in the Chinese Clinical Trial Registry with registration (ChiCTR2200064585).

### 2.2. Sample size calculation

The sample size was determined using the life orientation test revised (LOT-R) score as the primary outcome measure. Power analysis conducted with PASS version 15 indicated that a minimum of 22 participants per group was required to achieve adequate statistical power. Allowing for an anticipated 20% dropout rate, the required sample size was adjusted to at least 27 participants per group. Considering the annual number of newly diagnosed HIV cases in our institution and to enhance both the reliability and generalizability of the findings, the maximum sample size per group was predefined as 42. This specification was documented in the study’s ethical approval materials and clinical trial registration records.

### 2.3. Inclusion and exclusion criteria

Participants were eligible for inclusion if they met the following criteria: confirmed diagnosis of HIV according to the *Chinese Guidelines for Diagnosis and Treatment of HIV (2018 Edition*), with diagnosis within the past 3 months; age ≥18 years; no receipt of psychological therapy or counseling within the previous 6 months; completion of at least primary education and the ability to communicate effectively via smartphone; and voluntary participation with signed informed consent. Exclusion criteria were: cognitive impairment that hindered participation in the intervention; coexisting malignant tumors; diagnosed psychiatric disorders or current use of psychotropic medications.

### 2.4. Randomization and allocation

A total of 84 eligible participants were randomly assigned in a 1:1 ratio to either the intervention group or the control group using a computer-generated random number table. Allocation concealment was maintained by employing sealed, opaque envelopes, each containing a card specifying the participant’s assigned group. To minimize bias, both participants and outcome assessors were blinded to group assignments throughout the study period.

### 2.5. Intervention program

#### 2.5.1. Control group

The control group was given routine health instruction and psychological guidance, including medication guidance, life guidance, opportunistic infection prevention, psychological counseling and other health education, by case managers through face-to-face (week 1) and WeChat (weeks 2–8) communication. The case managers answered questions for patients at any time. The intervention lasted for 8 weeks.

#### 2.5.2. Experimental group

##### 2.5.2.1. Formation of the intervention team

An intervention team was established, comprising 1 head nurse and 4 staff nurses. The head nurse served as the team leader, responsible for overall coordination and supervision of the intervention process. The 4 staff nurses implemented the positive psychology–based expressive writing intervention among newly diagnosed HIV patients. To ensure methodological consistency and intervention fidelity, a psychiatric nurse specialist provided standardized training for all team members, focusing on the core concepts, procedures, and communication techniques of the intervention.

##### 2.5.2.2. Development of the intervention protocol

Drawing upon the theoretical framework of positive psychology and the psychological characteristics of newly diagnosed HIV patients, a structured expressive writing program was developed. The intervention included 6 thematic modules designed to foster positive emotions, enhance self-reflection, and promote adaptive coping. These modules were: *positive events, gratitude, personal strengths, savoring life, recalling pleasant experiences,* and *cultivating positivity* (Table 1).

**Table 1:** The specific content of expressive writing intervention.

Time (week)	Content of intervention	Explication
1th	Positive events	Record 3 good things that happen every day and think about why they happen. Record every day for a week.
2th	Gratitude	Recall help in life, write a thank-you letter or call the other party to express gratitude. Finish once.
3th	Highlights	Summarize advantages, and record practices and results. Finish once.
4th	Life experience	Recall little things in life, learn to enjoy them, record and aftertaste the pleasant feeling every night, and compare it with the previous feeling. Record once a day for a week.
5th	Aftertaste of beauty	Recall good experiences, successes or unforgettable happy moments in life, and record them in a diary of 1–2 pages. Finish once.
6th	Positive progress	When there are negative thoughts or emotions, write down a thing that gives the feeling of happiness in detail, mobilizes positive emotions, and drives away negative thoughts. Three times a week.
7–8th	Repetition drill	Select tasks that you want to repeat most from those of the previous few weeks, and practice and record them in the next 2 weeks.

##### 2.5.2.3. Intervention procedure

In addition to the routine care received by the control group, participants in the experimental group underwent an 8-week expressive writing intervention grounded in positive psychology. The intervention was delivered through a hybrid approach combining face-to-face sessions and WeChat-based communication, facilitated by the 4 trained nurses.

During the first week, a face-to-face session was conducted in the outpatient clinic. Nurses introduced the full 8-week intervention framework, explained the objectives and expectations of each module, and guided patients through the first 30-minute writing exercise. Subsequent weekly sessions (weeks 2–8) were conducted either in person or via WeChat, each lasting approximately 30 minutes.

Nurses provided ongoing guidance, encouraging participants to complete each writing task and document their reflections either on paper or in electronic format. Participants were instructed to send photos or screenshots of their completed work to the nurses for review. If a participant failed to submit the task on time, the assigned nurse promptly followed up through personalized communication to assess barriers and offer support, ensuring timely task completion and adherence to the intervention schedule.

### 2.6. Evaluation indicators

General Information Questionnaire: Baseline demographic and clinical data were collected using a self-designed questionnaire, including gender, age, educational level, marital status, route of HIV infection, HIV viral load, and CD4^+^ T lymphocyte count.

Life Orientation Test Revised (LOT-R): The level of optimism was assessed using the LOT-R, one of the most widely applied instruments for measuring dispositional optimism. The scale comprises 6 items, each rated on a 5-point Likert scale ranging from 0 (“strongly disagree”) to 4 (“strongly agree”). Items 1, 3, and 6 are positively scored, while items 2, 4, and 5 are reverse scored. The total score represents the sum of all item scores, with higher scores indicating a greater degree of optimism. The Chinese version of the LOT-R has demonstrated satisfactory internal consistency, with a Cronbach α coefficient of 0.78, and good construct validity.^[11]^

Medical Coping Modes Questionnaire (MCMQ): Coping styles were measured using the Chinese version of the MCMQ revised by Shen Xiaohong et al The scale assesses 3 dimensions of coping: confrontation, avoidance, and yielding, comprising a total of 20 items.Confrontation: items 1, 2, 5, 10, 12, 15, 16, and 19; Avoidance: items 3, 7, 8, 9, 11, 14, and 17; Yielding: items 4, 6, 13, 18, and 20.Each item is rated on a 4-point Likert scale (1–4). Items 1, 4, 9, 10, 12, 13, 18, and 19 are reverse scored, while the remaining items are positively scored. The score ranges for the confrontation, avoidance, and yielding dimensions are 8 to 32, 7 to 28, and 5 to 20, respectively. A higher score in each dimension indicates a stronger tendency to adopt that specific coping style. The internal consistency coefficients for the 3 subscales are 0.69, 0.60, and 0.76, respectively, indicating acceptable reliability.^[12]^

### 2.7. Data collection

Data were collected at 2 time points: baseline (prior to intervention) and post-intervention (after 8 weeks). At baseline, both groups completed the general information questionnaire, the LOT-R, and the MCMQ. After the 8-week intervention period, both groups were reassessed using the LOT-R and MCMQ to evaluate changes in optimism and coping styles. All questionnaires were administered by trained nurses following standardized instructions to ensure data consistency and accuracy.

### 2.8. Statistical analysis

Data were entered and analyzed using SPSS version 26.0 (IBM Corp., Armonk). Two independent researchers performed double data entry and cross-checked all entries to minimize input errors.Categorical variables were summarized using frequencies and percentages, while continuous variables were expressed as medians and interquartile ranges 𝑀(𝑃25,𝑃75) due to non-normal distribution.Between-group comparisons of categorical variables were conducted using the chi-square (χ^2^) test, and non-normally distributed continuous variables were compared using the Mann–Whitney *U* test. Within-group comparisons before and after intervention were analyzed using the Wilcoxon signed-rank test.All statistical tests were 2-tailed, and a *P*-value <.05 was considered statistically significant.

## 3. Results

### 3.1. Comparison of baseline characteristics between the 2 groups

**Figure 1.**
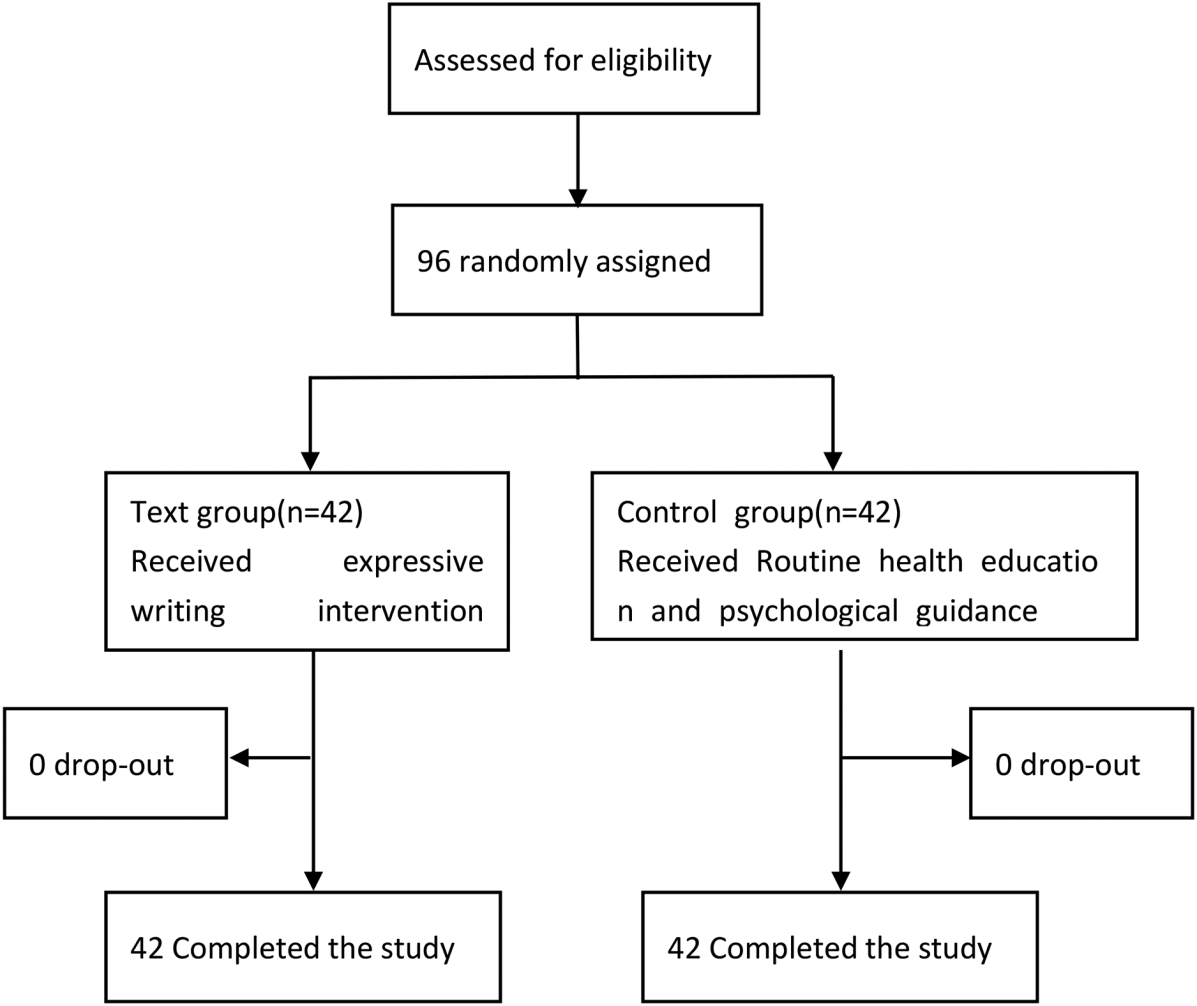
CONSORT flow diagram.

The CONSORT flow diagram for this study is shown in Figure 1. A total of 84 newly diagnosed HIV patients were enrolled and randomly assigned to either the experimental group (n = 42) or the control group (n = 42). All participants successfully completed the 8-week intervention, and no participants were lost to follow-up.

The complete retention of participants may reflect the strong healthcare needs and desire for professional support typically observed among individuals newly diagnosed with HIV. Participation in the study likely offered a sense of medical attention and psychological support, enhancing their motivation to remain engaged. In addition, the hybrid delivery model – combining face-to-face interaction with WeChat-based communication – lowered participation barriers and facilitated consistent adherence to the intervention throughout the study period.

In the experimental group, 41 participants were male and 1 was female, with a median age of 27.00 years (interquartile range [IQR]: 23.00–34.00). In the control group, 41 participants were male and 1 was female, with a median age of 30.00 years (IQR: 24.75–33.25). No statistically significant differences were observed in baseline demographic or clinical characteristics between the 2 groups (*P* >.05), confirming the comparability of the groups (Table 2).

**Table 2:** Basic clinical data.

Content	Classification	Text group (n = 42) (n,%)	Control group (n = 42) (n = 42)	Statistical value (χ^2^/Z)	*P*-value
Gender	Man	41 (97.62)	41 (97.62)	0.001	1.00
	Woman	1 (2.38)	1 (2.38)		
Age	20–29	26 (61.90)	20 (47.62)	4.38	.11
	30–39	14 (33.33)	14 (33.33)		
	≥40	2 (4.77)	8 (19.05)		
Education	Junior high school below	1 (2.38)	4 (9.52)	2.57	.28
	High school	20 (47.62)	22 (52.38)		
	University or above	21 (50.00)	16 (38.10)		
Marital status	Unmarried	39 (92.85)	35 (83.33)	4.12	.13
	Married	1 (2.38)	6 (14.29)		
	Divorce	2 (4.77)	1 (2.38)		
Route of infection	same sex	31 (73.81)	27 (64.28)	2.28	.32
	opposite sex	4 (9.52)	9 (21.43)		
	unknown	7 (16.67)	6 (14.29)		
HIV viral load (copies/mL)	≤100	3 (7.14)	2 (4.77)	−0.37	.72
	101~9999	9 (21.43)	12 (28.57)		
	≥10,000	30 (71.43)	28 (66.66)		
CD4 + T lymphocyte count (cells/μl)	≤200	8 (19.05)	11 (26.19)	−0.16	.87
	201–350	15 (35.71)	14 (33.33)		
	351–499	15 (35.71)	8 (19.05)		
	≥500	4 (9.53)	9 (21.43)		

HIV = human immunodeficiency virus.

### 3.2. LOT-R scores

Before the intervention, the LOT-R scores were at the same level in the 2 groups, with no statistically significant difference (*P* = .08). After the 8-week intervention, the scores were still not statistically significant in the control group (*P* = .21), but in the test group, the scores were significantly higher than those before the intervention (*P* <.001). The difference in scores between the 2 groups was statistically significant after 8 weeks of intervention (*P* = .02) (Table 3).

**Table 3:** Comparison of LOT-R score between the 2 groups.

Group	n	Baseline (*M*[*P*_25_, *P*_75_])	After intervention (*M*[*P*_25_, *P*_75_])	*Z* value	*P*-value
Test group	42	13.00 (11.00, 16.00)	16.00 (15.00, 18.00)	−5.24	<.001
Control group	42	14.00 (13.00, 16.25)	15.00 (13.00, 17.00)	−1.25	.21
*Z* value	–	−1.74	−2.43	–	–
*P*-value	–	.08	.02	–	–

LOT-R = life orientation test revised.

### 3.3. MCMQ scores

The baseline of the facing dimension scores of MCMQ in the 2 groups was at the same level (*P* = .08). After the 8-week intervention, the scores were still at the same level in the control group, with no statistically significant difference (*P* = .10). In the test group, however, the facing dimension scores of MCMQ were significantly higher than those before the intervention (*P* <.05), revealing that the coping style of individuals in the test group became more active through the intervention. The difference in the facing dimension scores between the 2 groups was statistically significant after 8 weeks of intervention (*P* <.001) (Table 4).

**Table 4:** Comparison of face dimension scores of MCMQ between the 2 groups.

Group	n	Baseline (*M*[*P*_25_, *P*_75_])	After intervention (*M*[*P*_25_, *P*_75_])	*Z* value	*P*-value
Test group	42	15.00 (14.00, 18.00)	16.50 (15.00, 18.25)	−2.10	.04
Control group	42	14.50 (13.00,16.00)	14.00 (13.00,16.00)	−1.63	.10
*Z* value	–	−1.73	−3.94	–	–
*P*-value	–	.08	<.001	–	–

MCMQ = Medical Coping Modes Questionnaire.

The baseline of the avoiding dimension scores of MCMQ in the 2 groups was at the same level, with no statistically significant difference (*P* = .82). After the 8-week intervention, the scores were still at the same level in the control group, with no statistically significant difference (*P* = .46). In the test group, however, the avoiding dimension scores of MCMQ were significantly lower than those before the intervention, with statistically significant differences (*P* <.05), indicating that the negative coping style of individuals in the test group was reduced after the intervention. The difference in the avoiding dimension scores between the 2 groups was statistically significant after 8 weeks of intervention (*P* = .00) (Table 5).

**Table 5:** Comparison of avoiding dimension scores of MCMQ between the 2 groups.

Group	n	Baseline (*M*([_25_, *P*_75_])	After intervention (*M*[*P*_25_, *P*_75_])	*Z* value	*P*-value
Test group	42	17.00 (15.00, 18.25)	15.00 (14.00, 16.00)	−2.94	.00
Control group	42	17.00 (15.00, 19.00)	16.00 (15.00, 18.00)	−0.75	.46
*Z* value	–	−0.24	−3.28	–	–
*P*-value	–	.82	<.001	–	–

MCMQ = Medical Coping Modes Questionnaire.

The baseline of the yielding dimension scores of MCMQ in the 2 groups was at the same level (*P* = .17). After the 8-week intervention, the scores were still at the same level in the control group, with no statistically significant difference (*P* = .15). In the test group, however, the yielding dimension scores of MCMQ were significantly lower than those before the intervention, with statistically significant differences (*P* <.001), indicating that the negative coping style of individuals in the test group was reduced after the intervention; The difference of the yielding dimension scores between the 2 groups was statistically significant (*P* = .00) (Table 6).

**Table 6:** Comparison of yielding dimension scores of MCMQ between the 2 groups.

Group	n	Baseline (*M*[*P*_25_, *P*_75_])	After intervention (*M*[*P*_25_, *P*_75_])	*Z* value	*P*-value
Test group	42	12.00 (10.00, 14.00)	9.00 (6.75, 10.25)	−4.96	<.001
Control group	42	11.00 (10.00, 13.00)	10.50 (9.00, 12.00)	−1.44	.15
*Z* value	–	−1.38	−3.23	–	–
*P*-value	–	.17	<.001	–	–

MCMQ = Medical Coping Modes Questionnaire.

## 4. Discussion

### 4.1. Enhancement of optimism through an expressive writing–based positive psychology intervention

The present study demonstrated that an 8-week expressive writing–based positive psychology intervention significantly enhanced optimism among newly diagnosed individuals with HIV. This result aligns with findings by Fair,^[13]^ who reported that expressive writing grounded in positive psychology promotes personal growth, alleviates feelings of isolation, and fosters positive emotional experiences among people living with HIV. In the current intervention, modules focusing on gratitude, savoring positive experiences, and cultivating optimism guided participants to recognize and reflect on positive life events. By encouraging awareness of uplifting aspects of daily life, the intervention helped patients build psychological resources, reframe maladaptive cognitions, achieve emotional equilibrium, and foster a dispositional tendency toward optimism. These outcomes are consistent with Fredrickson Broaden-and-Build Theory of Positive Emotions,^[14]^ which posits that positive emotions expand individuals’ thought–action repertoires and build enduring psychological and social resources.^[15]^ Through structured expressive writing, participants reconstructed their illness-related cognitive frameworks, shifting focus from the negative implications of HIV infection toward potential positive life changes. Consequently, the intervention empowered individuals to recognize personal strengths, mobilize social support, and attend to controllable aspects of their future – ultimately mitigating self-stigmatization and enhancing psychological well-being.

### 4.2. Improvement in coping styles through expressive writing–based positive psychology

The findings further indicated that expressive writing–based positive psychology effectively improved coping styles among newly diagnosed HIV patients. This observation is consistent with prior evidence suggesting that such interventions enhance coping flexibility and adaptive strategies across diverse populations, including university students.^[16]^ According to the Broaden-and-Build Theory, positive emotions not only broaden immediate thought–action repertoires but also facilitate the development of long-term cognitive, psychological, and social resources.^[17]^ These resources enhance resilience, optimism, and adaptability, enabling individuals to better manage future stressors. In the present study, participants were guided to describe positive experiences, recall uplifting events, and reflect on pleasant emotions, thereby stimulating positive affect and broadening cognitive flexibility. From a cognitive–behavioral perspective, positive emotions increase the breadth of cognitive and behavioral repertoires, enabling more flexible and open responses to challenges.^[18]^ The accumulation of such emotions contributes to the formation of enduring adaptive resources. Moreover, positive affect and positive behavior reinforce each other; engagement in positive activities actively elicits positive emotions.^[19]^ Through expressive writing, participants explored personal strengths and positive life experiences, activated constructive emotional states, and reconstructed their illness-related cognitions. These psychological shifts promoted active engagement in post-diagnosis life reconstruction and reduced reliance on maladaptive coping strategies.

### 4.3. Limitations

Several limitations should be acknowledged. First, reflecting the national male-to-female HIV infection ratio (approximately 3.8:1), the study sample consisted predominantly of men (97.6% in both groups), which may limit the generalizability of findings to female patients. Second, the intervention relied on participants’ familiarity with WeChat and other digital platforms, resulting in a younger, more technologically literate sample and potentially limiting applicability to older populations. Third, as a single-center study with a relatively small sample size and short-term follow-up, the long-term effects of the intervention could not be assessed. Future research should employ multicenter randomized controlled trials with larger, more diverse samples and extended follow-up durations to evaluate the sustainability of intervention effects.

### 4.4. Implications for clinical practice and future research

Newly diagnosed individuals with HIV often experience profound psychological distress stemming from stigma, disrupted support networks, and fear of disclosure. Such distress can impair treatment adherence and, in extreme cases, precipitate self-harming behaviors, thereby adversely affecting prognosis. The current findings suggest that expressive writing–based positive psychology interventions effectively enhance optimism and promote adaptive coping, underscoring their potential role in psychological and behavioral care for this population.Clinicians should closely monitor the mental health of newly diagnosed HIV patients and consider incorporating expressive writing–based positive psychology into standard care as a feasible, low-cost, and easily implemented strategy for emotional regulation. Owing to its simplicity, convenience, and strong patient acceptability, this approach holds promise for integration into clinical and community-based HIV care programs.

## 5. Conclusion

An 8-week expressive writing–based positive psychology intervention significantly improved optimism and coping strategies among newly diagnosed HIV patients. Nonetheless, the single-center design, limited sample size, and short follow-up period may restrict the external validity of these findings. Future multicenter studies with larger, heterogeneous samples and longitudinal follow-up of at least 12 months are warranted. Incorporating both psychosocial and physiological indicators will further elucidate the long-term efficacy and underlying mechanisms of expressive writing–based positive psychology interventions in individuals living with HIV.

## Author contributions

**Conceptualization:** Juan Tan, Jing Yang.

**Formal analysis:** Juan Tan, Chuntao Wu.

**Investigation:** Shiping Feng, Qian Wen, Peisha Du, Chuntao Wu.

**Project administration:** Qian Wen.

**Supervision:** Jing Yang.

**Writing – original draft:** Shiping Feng.

**Writing – review & editing:** Jing Yang.
